# Analytic Modeling of Neural Tissue: I. A Spherical Bidomain

**DOI:** 10.1186/s13408-016-0041-1

**Published:** 2016-09-09

**Authors:** Benjamin L. Schwartz, Munish Chauhan, Rosalind J. Sadleir

**Affiliations:** School of Biological and Health Systems Engineering, Arizona State University, 501 E Tyler Mall, Tempe, AZ 85287-9709 USA

**Keywords:** Bidomain, Analytic modeling

## Abstract

Presented here is a model of neural tissue in a conductive medium stimulated by externally injected currents. The tissue is described as a conductively isotropic bidomain, i.e. comprised of intra and extracellular regions that occupy the same space, as well as the membrane that divides them, and the injection currents are described as a pair of source and sink points. The problem is solved in three spatial dimensions and defined in spherical coordinates $(r,\theta,\phi )$. The system of coupled partial differential equations is solved by recasting the problem to be in terms of the membrane and a monodomain, interpreted as a weighted average of the intra and extracellular domains. The membrane and monodomain are defined by the scalar Helmholtz and Laplace equations, respectively, which are both separable in spherical coordinates. Product solutions are thus assumed and given through certain transcendental functions. From these electrical potentials, analytic expressions for current density are derived and from those fields the magnetic flux density is calculated. Numerical examples are considered wherein the interstitial conductivity is varied, as well as the limiting case of the problem simplifying to two dimensions due to azimuthal independence. Finally, future modeling work is discussed.

## Introduction

The purpose of this paper is to model the electric potentials in and around a finite volume of excitable tissue that result from externally applied injection current. Our motivation toward quantitative understanding of the distributed electrophysiology of excitable tissue is due to the emergence of magnetic resonance electrical impedance tomography (MREIT) [1]. The contrast in MREIT—as well as in another MR technique, Electrical Properties Tomography (EPT) [2]—depends on the electrical property distribution throughout the region of interest. Briefly, in an MREIT scan, current is injected into an object in concert with the pulse sequence of an MRI scanner. This current will induce a magnetic field [3] whose distribution throughout the entire region can be captured via the phase component of the reconstructed MR images. Electrical conductivity maps may then be constructed from the phase data using the Laplacian of the z component of the induced magnetic field, $\nabla^{2} B_{z}$ [4, 5]. MREIT has already shown clinical promise, e.g. lesion characterization [6], but it is the possibility of monitoring brain activity with MREIT [7] that especially motivates this study. If MREIT is to be used to detect neural activity it is useful to estimate the influence of MREIT imaging currents on both active and passive tissues. Therefore, we have constructed from first principles an analytic mathematical model of tissue stimulated by injection currents, not unlike that of an MREIT scan.

Excitable tissues are comprised of cells, discrete units through which electric signals may propagate via action potentials [8]. While many have studied and modeled the behavior of individual cells in both sub- and supra-threshold conditions, it is also very important to understand excitability behavior at the tissue level. This approach has been particularly useful in understanding cardiac activity [9]. The bidomain model [10], a generalization of the cable equation [11], has been employed in this area avoiding the discrete constructs of tissue, assuming instead a continuum of two domains, intra- and extracellular, connected by a membrane and that occupy the same volume [12]. Each domain represents an average, then, of all its individual components. MR imaging also necessarily involves averaging over tissues. If we seek to image neural activity using MREIT it is convenient to use a geometrically simple model to predict changes in these images created by neural activity.

Many authors have modeled excitable tissue with the bidomain equations, choosing the coordinate system that most closely resembles the tissue geometry. In circular cylindrical coordinates, Altman and Plonsey modeled a bundle of nerves as an infinite cylinder in an infinite conducting bath, studying first the steady state [13] and transient stimulation [14]. In the former they incrementally increased the realism of their model, going from an isotropic monodomain to an anisotropic bidomain, while in the latter they investigated the effect of fiber diameter on stimulation and impulse propagation. Henriquez et al. [15–17] and Trayanova et al. [18] investigated the merits of assuming a single fiber vs. a bundle, i.e. bidomain, of fibers when modeling an infinite cylinder of tissue excited by either a disk or a line source. They showed that the single fiber core conductor model is not an unreasonable approximation of the control region of a large bundle of fibers, but loses its validity toward the periphery of the bundle and is entirely unsatisfactory for small bundles. Plonsey and Barr showed in a two dimensional rectangular framework, except for special cases, the bidomain approach to modeling tissue electrophysiology is not a mere generalization of one dimensional cable theory [19, 20]. They found that current flowed very differently in isotropic tissue compared to anisotropic tissue with unequal anisotropy ratios. Roth gave approximate analytic solutions to the problem of bisyncytia with unequal anisotropy ratios [21], using rectangular coordinates. His perturbation method involved expansion in a parameter defined through the anisotropy ratios. He considered two sources: an expanding wave front that was approximated with a step function, and a point source. Trayanova et al. considered the case of bidomain tissue in a uniform electric field, modeling the heart as a sphere of anisotropic tissue with a core of blood [22]. The uniform field meant that they could assume azimuthal independence, leaving only a two dimensional problem in the spherical coordinates *r* and *θ*. Heretofore none has studied a three spatial dimension bidomain problem in spherical coordinates.

Our present study is motivated by the need to understand the effect on MREIT images of excitable tissue—specifically, a ganglion excised from the abdomen of a sea slug (*Aplysia californica*)—affected by injection currents injected through electrodes set into the boundary of its artificial sea water bath [7]. We develop a model that is a dramatic simplification of the actual experiment but which still is novel for its generalization to three spherical dimensions. In and of itself this model will depict basic electrophysiological phenomena and can act as a standard against which numeric simulations such as finite element models (FEM) are held, lending credibility to those in concurrence. Seen in a broader context, this work can serve as the foundation for more and more sophisticated analytic modeling, e.g. nonlinear transmembrane currents and mixed boundary conditions.

In this first study of three dimensional analysis of distributed neural tissue we model the *Aplysia* abdominal ganglion (AG), known to be electrically coupled by gap junctions [23], as an isotropic bidomain sphere, the artificial sea water bath as an infinite isotropic conducting medium, and the injection currents as source as sink points. We assume isotropic conductivity here for simplicity. However, anisotropy may be the subject of future work, as active tissue is generally anisotropic.

## Problem Formulation

### Geometry

**Fig. 1:**
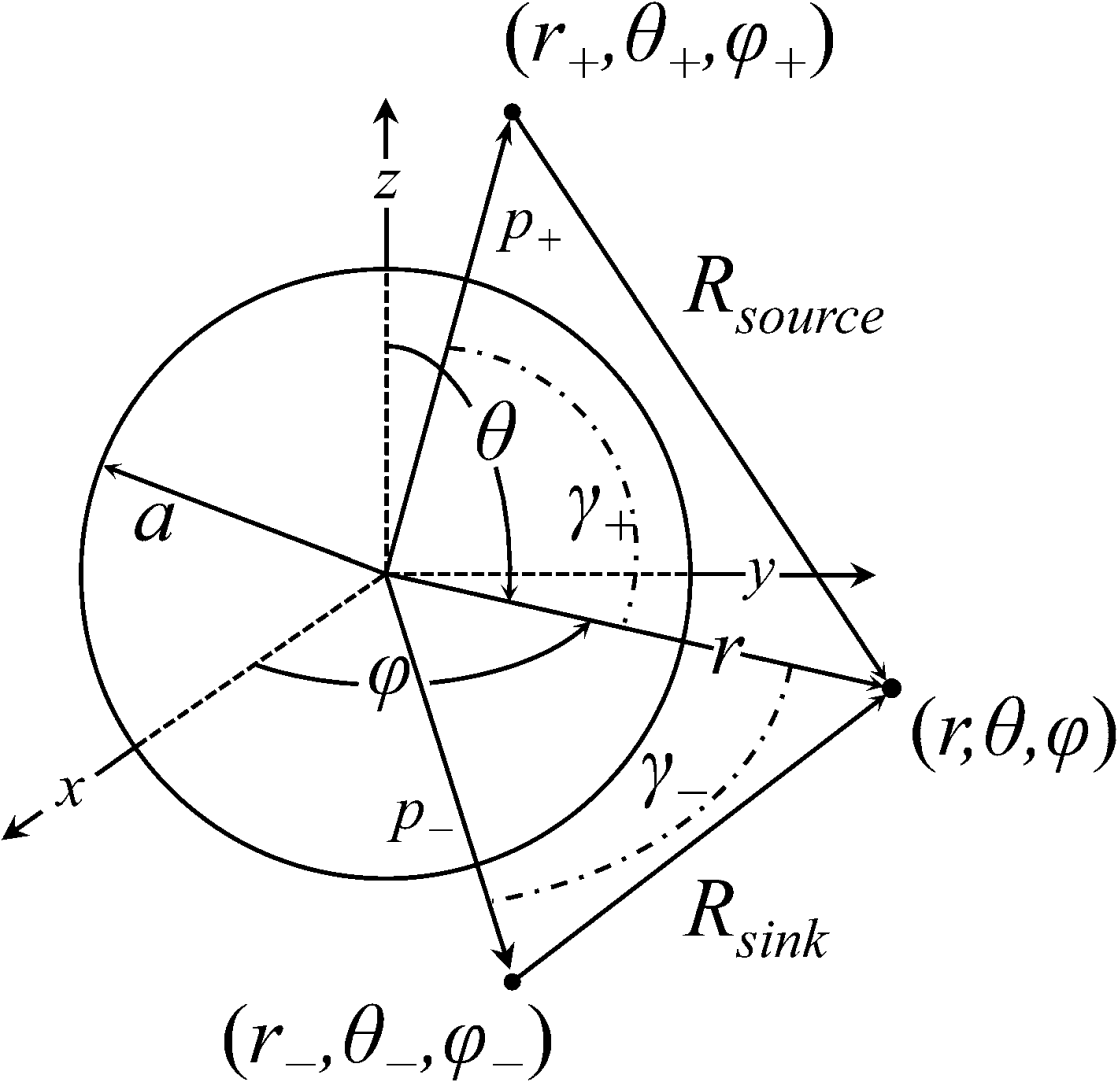
Sphere with radius $r=a$ in an infinite conducting medium with current points source and sink at $\mathbf {p}_{+}=(r_{+},\theta _{+},\varphi_{+})$ and $\mathbf {p}_{-}=(r_{-},\theta_{-},\varphi_{-})$, respectively. *The dot-dashed curves* labeled as *γ* are the angles between the points’ position vectors and that of a field point $\mathbf {r}=(r,\theta,\varphi)$

Let there be given a sphere of isotropic excitable tissue in a uniform isotropic infinite conducting bath which also contains a point current source and a point current sink. We shall consider this problem in terms of spherical coordinates $(r,\theta,\varphi)$ [24]. The sphere of tissue, whose radius is $r=a$, has its center at the origin. The current source and sink points are distances $\mathbf {p}_{+}=(r_{+},\theta_{+},\varphi_{+})$ and $\mathbf {p}_{-}=(r_{-},\theta_{-},\varphi_{-})$, respectively, from the origin, as shown in Fig. 1. The current source and sink are in the conducting bath, not in the tissue, i.e. $a< r_{+},r_{-}$. The segments $R_{\mathrm{source}}$ and $R_{\mathrm{sink}}$ are the respective distances from the source and sink to any field point with position vector $\mathbf {r}=(r,\theta,\varphi)$. The angles $\gamma _{+}$ and $\gamma_{-}$, drawn with a dot-dashed line in Fig. 1 are between $\mathbf {p}_{+}$ and **r**, and $\mathbf {p}_{-}$ and **r**, respectively.

### Bidomain Tissue

The tissue is modeled as a bidomain: two regions—intracellular and extracellular—that occupy the same volume along with the membrane that separates them. Any transmembrane current must be either from the intracellular region to the extracellular region, $I_{m}=\nabla\cdot \mathbf {J}_{i}$, or vice versa, $I_{m}=-\nabla\cdot \mathbf {J}_{o}$ [10]. From Ohm’s law, $\mathbf {J}=\mathbf {E}/ \rho$, where **E** is electric field strength and *ρ* is resistivity [3]. Assuming that **E** is quasistatic [25] and, further, that there is no tissue capacitance, we may express **E** in terms of scalar potentials, *ϕ*, i.e. $\mathbf {E}=-\nabla\phi$. Thus we arrive at the bidomain equations,

1a $$\begin{aligned} \nabla^{2}\phi_{i}&=I_{m} \rho_{i}, \end{aligned}$$

1b $$\begin{aligned} \nabla^{2}\phi_{o}&=-I_{m} \rho_{o}, \end{aligned}$$

where the bidomain potentials, $\phi_{i}$ and $\phi_{o}$, are the intra- and extracellular potentials, respectively, and $\rho_{i}$ and $\rho_{o}$ are the corresponding resistivities. Throughout this analysis we assume the membrane to be passive resistor which makes $I_{m}$ depend upon the difference between $\phi_{i}$ and $\phi_{o}$,

2 $$ I_{m}= (\phi_{i}-\phi_{o} ) \frac{\beta}{R_{m}}, $$

where $R_{m}$ is the membrane resistance times unit area and *β* is the ratio of the membrane’s surface area to volume of the cell. Equations (1a)-(1b) are coupled and must be un-coupled to solve for $\phi_{i}$ and $\phi_{o}$ by recasting the system in terms of the transmembrane potential, $V_{m}$, and the monodomain potential, *ψ* [26],

3a $$\begin{aligned} V_{m}&=\phi_{i}-\phi_{o}, \end{aligned}$$

3b $$\begin{aligned} \psi&=\frac{\rho_{o}}{\rho_{o}+\rho_{i}}\phi_{i}+\frac{\rho_{i}}{\rho_{o}+\rho_{i}} \phi_{o}. \end{aligned}$$

The bidomain potentials are, then, given as

4a $$\begin{aligned} \phi_{i}&=\frac{\rho_{i}}{\rho_{o}+\rho_{i}}V_{m}+\psi, \end{aligned}$$

4b $$\begin{aligned} \phi_{o}&=-\frac{\rho_{o}}{\rho_{o}+\rho_{i}}V_{m}+\psi. \end{aligned}$$

To solve for $V_{m}$, let us subtract Eq. (1b) from Eq. (1a)

5 $$ \nabla^{2}\phi_{i}-\nabla^{2} \phi_{o}=I_{m} ( \rho_{i} +\rho_{o} ), $$

and then insert Eq. (2),

6 $$ \nabla^{2} ( \phi_{i}-\phi_{o} )= \frac{\phi_{i}-\phi_{o}}{\rho_{m}} ( \rho_{i} +\rho_{o} ), $$

where $\rho_{m}=R_{m}/\beta$ is the membrane resistance times unit volume. If we define a length constant, $\lambda=\sqrt{\rho_{m}/ ( \rho_{i} +\rho_{o} )}$, from Eq. (3a) we can immediately see that $V_{m}$ satisfies the scalar Helmholtz equation,

7 $$ \nabla^{2}V_{m}-\frac{V_{m}}{\lambda^{2}}=0. $$

To find a relationship that only involves *ψ*, let us apply the Laplacian operator to Eq. (3b):

8 $$ \nabla^{2}\psi=\frac{\rho_{o}}{\rho_{i} +\rho_{o}}\nabla^{2} \phi_{i}+\frac{\rho _{i}}{\rho_{i} +\rho_{o}}\nabla^{2}\phi_{o}. $$

When we put our expressions from Eqs. (1a)-(1b) for $\nabla^{2}\phi_{i}$ and $\nabla^{2}\phi_{o}$ into the right side of Eq. (8), the two terms on that side add to 0, leaving us with the Laplace equation through *ψ*,

9 $$ \nabla^{2}\psi=0. $$

### Infinite Medium

External to the tissue the potential, $\phi_{e}$, is given by

10 $$ \phi_{e}=\phi_{\mathrm{bath}}+\phi_{\mathrm{source}}+ \phi_{\mathrm{sink}}, $$

where $\phi_{\mathrm{source}}$ and $\phi_{\mathrm{sink}}$ are the fields due to the current point source and current point sink, respectively, and $\phi _{\mathrm{bath}}$ is the secondary field [27] which satisfies the Laplace equation,

11 $$ \nabla^{2}\phi_{\mathrm{bath}}=0. $$

## Solutions

### Transmembrane Potential

The scalar Laplacian of a function *f* is defined as the divergence of the gradient of *f* [24]

12 $$ \nabla^{2}f\equiv\nabla\cdot\nabla f= \sqrt{g} \sum _{i=1}^{3} \frac{\partial }{\partial u_{i}} \biggl( \frac{\sqrt{g}}{g_{i}} \frac{\partial f}{\partial u_{i}} \biggr), $$

where, in spherical coordinates, $u_{1}=r$, $u_{2}=\theta$, $u_{3}=\varphi$, $g_{1}=1$, $g_{2}=r^{2}$, $g_{3}=r^{2}\sin^{2}(\theta)$, and $\sqrt{g}=r^{2}\sin (\theta )$, whence we arrive at the familiar expression [24]

13 $$ \nabla^{2} f = \frac{\partial^{2} f}{\partial r^{2}}+ \frac{2}{r} \frac {\partial f}{\partial r}+ \frac{1}{ r^{2}} \frac{\partial^{2} f}{\partial \theta^{2}} + \frac{\cot(\theta)}{r^{2}} \frac{\partial f}{\partial\theta }+ \frac{1}{r^{2} \sin^{2} (\theta)} \frac{\partial^{2} f}{\partial\varphi^{2}}. $$

If we apply Eq. (13) to Eq. (7) and assume a product solution of the transmembrane potential $V_{m}=R(r)\varTheta(\theta)\varPhi(\varphi)$, we can use the method of separation of variables to get three independent, linear, ordinary, second order, differential equations [24],

14a $$\begin{gathered} \frac{\partial^{2}R(r)}{\partial r^{2}}+\frac{2}{r}\frac{\partial R(r)}{\partial r}- \biggl( \frac{1}{\lambda^{2}} +\frac{\alpha}{r^{2}} \biggr) R(r)=0, \end{gathered}$$

14b $$\begin{gathered} \frac{\partial^{2}\varTheta(\theta)}{\partial\theta^{2}}+\cot(\theta) \frac {\partial\varTheta(\theta)}{\partial\theta}+ \biggl( \alpha-\frac{\nu ^{2}}{\sin^{2}(\theta)} \biggr) \varTheta(\theta)=0, \end{gathered}$$

14c $$\begin{gathered} \frac{\partial^{2}\varPhi(\varphi)}{\partial\phi^{2}}+\nu^{2} \varPhi(\varphi)=0, \end{gathered}$$

where $\alpha=\mu(\mu+1)$. Equation (14a) admits two solutions, $i_{\mu}$ and $k_{\mu}$, the modified spherical Bessel functions of the first and second kind of order *μ*, respectively [28]. We only use $i_{\mu}$, however, because the domain includes the origin, where $k_{\mu}$ is singular. The transmembrane potential, then, is

15 $$ V_{m}(r,\theta,\varphi)=\sum _{\mu=0}^{\infty} \sum_{\nu=-\mu}^{\mu} a_{\mu \nu} i_{\mu} \biggl( \frac{r}{\lambda} \biggr) Y_{\mu}^{\nu} (\theta ,\varphi), $$

where $Y_{\mu}^{\nu}$ is the tesseral spherical harmonic [29] of degree *μ* and order *ν*, and $a_{\mu\nu}$ is the coefficient determined from the boundary conditions.

### Monodomain Potential

For the monodomain potential, let us once again assume a product solution $\psi(r,\theta,\varphi)=\mathscr{R}(r)\varTheta(\theta)\varPhi (\varphi)$. Applying Eq. (13), we separate Eq. (9) into three equations. The radial equation is [24]

16 $$ \frac{\partial^{2}\mathscr{R}(r)}{\partial r^{2}}+\frac{2}{r}\frac{\partial \mathscr{R}(r)}{\partial r}- \frac{\alpha}{r^{2}} \mathscr{R}(r)=0, $$

and the equations through $\varTheta(\theta)$ and $\varPhi(\varphi)$ are the same as in Eqs. (14b) and (14c), respectively. Like Eq. (14a), Eq. (16), too, admits two solutions, $r^{\mu}$ and $r^{-\mu-1}$. For the sake of analyticity, clearly we must omit $r^{-\mu -1}$, making the solution for the monodomain potential

17 $$ \psi(r,\theta,\varphi)=\sum_{\mu=0}^{\infty} \sum_{\nu=-\mu}^{\mu} b_{\mu\nu} r^{\mu} Y_{\mu}^{\nu} (\theta,\varphi), $$

where $b_{\mu\nu}$ is the a coefficient to be determined by the boundary conditions.

### External Potential

The potential from the external bath also satisfies the Laplace equation, but its domain does not include the origin, so we may immediately write its solution as

18 $$ \phi_{\mathrm{bath}}(r,\theta,\varphi)=\sum _{\mu=0}^{\infty} \sum_{\nu=-\mu}^{\mu } c_{\mu\nu} r^{-\mu-1} Y_{\mu}^{\nu} (\theta,\varphi), $$

where $c_{\mu\nu}$ is determined by the boundary conditions. The potentials due to the current source and sink points with magnitude $I_{o}$ are given as [10]

19a $$\begin{aligned} \phi_{\mathrm{source}}&=\frac{I_{o} \rho_{e}}{4 \pi R_{\mathrm{source}}} , \end{aligned}$$

19b $$\begin{aligned} \phi_{\mathrm{sink}}&=-\frac{I_{o} \rho_{e}}{4 \pi R_{\mathrm{sink}}}, \end{aligned}$$

where $\rho_{e}$ is the resistivity of the conducting bath, and $R_{\mathrm{source}}$ and $R_{\mathrm{sink}}$ are the distances from their respective points to any point $(r,\theta,\varphi)$ in the problem domain, shown in Fig. 1. To satisfy the boundary conditions, $\phi_{\mathrm{source}}$ and $\phi_{\mathrm{sink}}$ must be written in terms of *r*, *θ*, and *φ*, the derivation of which can be found in the Appendix.

## Boundary Conditions

We have three boundary conditions [30] at the tissue-bath interface, where $r=a$, through which we will determine the unknown coefficients, $a_{\mu\nu}$, $b_{\mu\nu}$, and $c_{\mu\nu}$. They are continuity of external and extracellular potentials,

20 $$ \phi_{e}(a,\theta,\varphi)=\phi_{o}(a, \theta,\varphi), $$

continuity of normal current between bath and interstitium,

21 $$ \rho_{e}^{-1}\frac{\partial\phi_{e}(r,\theta,\varphi)}{\partial r}\bigg|_{r=a}= \rho_{o}^{-1}\frac{\partial\phi_{o}(r,\theta ,\varphi)}{\partial r}\bigg|_{r=a}, $$

and no intracellular normal current

22 $$ \rho_{i}^{-1}\frac{\partial\phi_{i}(r,\theta,\varphi)}{\partial r}\bigg|_{r=a}=0, $$

whose solutions yield

23a $$\begin{aligned} a_{\mu\nu}={}&I_{o} (-1)^{\nu} \mu a^{\mu+1}(r_{+}r_{-})^{-\mu-1}\rho_{e} \rho_{o} ( \rho_{i}+\rho_{o})p (2\mu+1)^{-1} q^{-1}, \end{aligned}$$

23b $$\begin{aligned} b_{\mu\nu}={}&I_{o} (-1)^{\nu+1} (r_{+}r_{-})^{-\mu-1} \rho_{e} \rho_{i} \rho_{o} (2 \mu+1)^{-1} q^{-1} \\ &{}\times p \biggl( \lambda\mu i_{\mu} \biggl( \frac{a}{\lambda} \biggr)+a i_{\mu+1} \biggl( \frac{a}{\lambda} \biggr) \biggr), \end{aligned}$$

23c $$\begin{aligned} c_{\mu\nu}={}&I_{o} (-1)^{\nu} \mu a^{2\mu+1}(r_{+}r_{-})^{-\mu-1} \rho_{e} (2\mu +1)^{-1} q^{-1} \\ &{}\times\biggl( r_{-}^{\mu+1}\rho_{0} \biggl( a \rho_{i} i_{\mu+1} \biggl( \frac{a}{\lambda} \biggr) + \lambda \mu (\rho_{i}+\rho_{o}) i_{\mu} \biggl( \frac{a}{\lambda} \biggr) \biggr) \\ &{}\times \bigl( Y_{\mu}^{-\nu}(\theta_{+},\varphi_{+})-Y_{\mu}^{-\nu}( \theta_{-},\varphi_{-}) \bigr) \\ &{}-\rho_{e}(\rho_{i}+\rho_{o}) \biggl( \lambda \mu i_{\mu} \biggl( \frac{a}{\lambda} \biggr)+a i_{\mu+1} \biggl( \frac{a}{\lambda} \biggr) \biggr) \\ &{}\times \bigl( r_{-}^{\mu+1}Y_{\mu}^{-\nu}(\theta_{+}, \varphi_{+})-r_{+}^{\mu+1}Y_{\mu}^{-\nu }(\theta_{-},\varphi_{-}) \bigr)\biggr), \end{aligned}$$

where

24 $$\begin{aligned} p =& \bigl( (\mu+1) r_{+}^{\mu+1}+\mu r_{-}^{\mu+1} \bigr) Y_{\mu}^{-\nu }(\theta_{-},\varphi_{-}) \\ &{}-(2\mu+1)r_{-}^{\mu+1}Y_{\mu}^{-\nu}(\theta _{+}, \varphi_{+}) \end{aligned}$$

and

25 $$\begin{aligned} q =& \lambda\mu (\rho_{i}+\rho_{o} ) \bigl( \mu\rho_{e}+(\mu+1)\rho _{o} \bigr) i_{\mu} \biggl( \frac{a}{\lambda} \biggr) \\ &{}-a i_{\mu+1} \biggl( \frac{a}{\lambda} \biggr) \bigl(\mu \rho_{e} (\rho _{i}+\rho_{o} )+(\mu+1) \rho_{i}\rho_{o} \bigr), \end{aligned}$$

completely determining all potential fields.

## Current Densities

Current density is proportional to the negative gradient of the scalar electric potential, $\mathbf {J}=-\rho^{-1}\nabla\phi$. The gradient of a function *f* is given as [31]

26 $$ \nabla f = \sum_{i=1}^{3} \frac{1}{\sqrt{g_{i}}}\frac{\partial f}{\partial u_{i}} \mathbf{a}_{i}. $$

In spherical coordinates $u_{i}$ and $g_{i}$ are the same as in Eq. (12) and the unit vectors are $\mathbf{a}_{1}=\mathbf {r}$, $\mathbf{a}_{2}=\boldsymbol{\theta}$, and $\mathbf{a}_{3}=\boldsymbol {\varphi}$ which gives us

27 $$ \nabla f = \frac{\partial f}{\partial r}\mathbf{r}+\frac{1}{r} \frac {\partial f}{\partial\theta}\boldsymbol{\theta}+\frac{1}{r \sin(\theta )}\frac{\partial f}{\partial\varphi} \boldsymbol{\varphi}. $$

Applying Eq. (27) to Eqs. (4a)-(4b) and (10) and dividing by the resistivities of their respective domains gives us expressions for the current densities throughout our problem. They are

28 $$\begin{aligned} \mathbf {J}_{i}(r,\theta,\varphi) =& -\biggl(\frac{a_{\mu\nu}}{\rho_{o}+\rho_{i}} \biggl( \mu\frac{\lambda}{r}i_{\mu} \biggl(\frac{r}{\lambda} \biggr)+i_{\mu+1} \biggl(\frac{r}{\lambda} \biggr) \biggr) \\ &{}+\frac{\mu}{\rho_{i}}b_{\mu\nu}r^{\mu-1}\biggr) Y_{\mu}^{\nu} (\theta,\varphi ) \mathbf{r} \\ &{}-\biggl( \biggl( \frac{a_{\mu\nu}}{\rho_{o}+\rho_{i}}\frac{1}{r}i_{\mu} \biggl( \frac {r}{\lambda} \biggr)+\frac{b_{\mu\nu}}{\rho_{i}}r^{\mu-1} \biggr) \\ &{}\times \bigl( \nu\cot (\theta ) Y_{\mu}^{\nu} (\theta, \varphi )+f (\mu,\nu ) e^{-i \varphi}Y_{\mu}^{\nu+1} (\theta , \varphi ) \bigr)\biggr) \boldsymbol{\theta} \\ &{}-\csc (\theta ) \biggl(\frac{a_{\mu\nu}}{\rho_{o}+\rho_{i}}\frac{1}{r}i_{\mu} \biggl(\frac {r}{\lambda} \biggr)+\frac{b_{\mu\nu}}{\rho_{i}}r^{\mu-1} \biggr)i\mu Y_{\mu}^{\nu} (\theta,\varphi ) \boldsymbol {\varphi}, \end{aligned}$$

29 $$\begin{aligned} \mathbf {J}_{o}(r,\theta,\varphi) =& \biggl(\frac{a_{\mu\nu}}{\rho_{o}+\rho_{i}} \biggl(\mu \frac{\lambda}{r}i_{\mu} \biggl(\frac{r}{\lambda} \biggr)+i_{\mu+1} \biggl(\frac{r}{\lambda} \biggr) \biggr) -\frac{\mu}{\rho_{o}}b_{\mu\nu}r^{\mu-1}\biggr) Y_{\mu}^{\nu} (\theta,\varphi ) \mathbf{r} \\ &{}+\biggl( \biggl( \frac{a_{\mu\nu}}{\rho_{o}+\rho_{i}}\frac{1}{r}i_{\mu} \biggl( \frac {r}{\lambda} \biggr)-\frac{b_{\mu\nu}}{\rho_{o}}r^{\mu-1} \biggr) \\ &{}\times \bigl( \nu\cot (\theta ) Y_{\mu}^{\nu} (\theta, \varphi )+f (\mu,\nu ) e^{-i \varphi}Y_{\mu}^{\nu+1} (\theta , \varphi ) \bigr)\biggr) \boldsymbol{\theta} \\ &{}+\csc (\theta ) \biggl( \frac{a_{\mu\nu}}{\rho_{o}+\rho_{i}}\frac{1}{r}i_{\mu} \biggl(\frac {r}{\lambda} \biggr)-\frac{b_{\mu\nu}}{\rho_{o}}r^{\mu-1} \biggr)i\mu Y_{\mu}^{\nu} (\theta,\varphi ) \boldsymbol {\varphi}, \end{aligned}$$

30 $$\begin{aligned} \mathbf {J}_{e}(r,\theta,\varphi) =& \biggl(\frac{c_{\mu\nu}}{\rho_{e} r^{2+\mu}}+ \frac{I_{0}}{2 \mu+1} \bigl(f_{-}(r) Y_{\mu}^{-\nu} (\theta_{-},\varphi_{-} ) \\ &{}-f_{+}(r) Y_{\mu }^{-\nu} (\theta_{+},\varphi_{+} )\bigr)\biggr) Y_{\mu}^{\nu} (\theta,\varphi ) \mathbf{r} \\ &{}- \biggl( \frac{c_{\mu\nu}}{\rho_{e} r^{2+\mu}}+ \frac{s}{r} \biggr) \bigl( \nu\cot ( \theta ) Y_{\mu}^{\nu} (\theta,\varphi ) +\tau e^{-i \varphi} Y_{\mu}^{\nu+1} (\theta,\varphi ) \bigr) \boldsymbol{\theta} \\ &{}- \biggl( \frac{c_{\mu\nu}}{\rho_{e} r^{2+\mu}}+ \frac{s}{r} \biggr) i \nu\csc (\theta ) Y_{\mu}^{\nu} (\theta,\varphi ) \boldsymbol{\varphi}, \end{aligned}$$

where

31 $$\begin{aligned} f_{+,-}(r)&= \textstyle\begin{cases} \mu\frac{r^{\mu-1}}{r_{+,-}^{\mu+1}}, & \text{if }r< r_{+,-}, \\ -(\mu+1)\frac{r_{+,-}^{\mu}}{r^{\mu+2}}, & \text{if }r>r_{+,-}, \end{cases}\displaystyle \end{aligned}$$

32 $$\begin{aligned} s&=\frac{I_{0}}{2\mu+1} \biggl(\frac{ (g_{+}^{< } )^{\mu}}{ (g_{+}^{>} )^{\mu+1}} Y_{\mu}^{-\nu} ( \theta_{+},\varphi_{+} )-\frac { (g_{-}^{< } )^{\mu}}{ (g_{-}^{>} )^{\mu+1}} Y_{\mu}^{-\nu } (\theta_{-}, \varphi_{-} ) \biggr), \end{aligned}$$

and

33 $$ \tau=\frac{\sqrt{\varGamma(\mu-\nu+1)} \sqrt{\varGamma(\mu+\nu+2)}}{\sqrt {\varGamma(\mu-\nu)} \sqrt{\varGamma(\mu+\nu+1)}}. $$

Here $g_{+,-}^{<}=\min(r,r_{+,-})$, $g_{+,-}^{>}=\max(r,r_{+,-})$, and *Γ* is the gamma function [32].

## Magnetic Flux Density

Here we summarize the theory behind our numeric calculation of the magnetic flux density, **B**, due to **J**. Within a current-carrying volume, **B** may be calculated from the Biot–Savart law [33],

34 $$ \mathbf {B}(\mathbf{r})=\frac{\mu_{0}}{4 \pi} \int_{V} \frac{\mathbf {J}(\mathbf {r}')\times|\mathbf{r}-\mathbf{r}'|}{ |\mathbf{r}-\mathbf{r}'|^{3}}\, dV', $$

thus every point in **B** requires integration over the entire current-carrying volume. Since the numerator of the integrand of Eq. (34) is a cross product the individual components of **B** in cartesian coordinates are given as

35a $$\begin{aligned} B_{x}&=\frac{\mu_{0}}{4 \pi} \int_{V} \frac{J_{y} (z-z')-J_{z} (y-y')}{ ( (x-x')^{2}+(y-y')^{2}+(z-z')^{2} )^{\frac{3}{2}}}\, dx'\, dy'\, dz', \end{aligned}$$

35b $$\begin{aligned} B_{y}&=\frac{\mu_{0}}{4 \pi} \int_{V} \frac{J_{z} (x-x')-J_{x} (z-z')}{ ( (x-x')^{2}+(y-y')^{2}+(z-z')^{2} )^{\frac{3}{2}}}\, dx'\, dy'\, dz', \end{aligned}$$

35c $$\begin{aligned} B_{z}&=\frac{\mu_{0}}{4 \pi} \int_{V} \frac{J_{x} (y-y')-J_{y} (x-x')}{ ( (x-x')^{2}+(y-y')^{2}+(z-z')^{2} )^{\frac{3}{2}}}\, dx'\, dy'\, dz'. \end{aligned}$$

In an MREIT scan we are only concerned with the component of the magnetic field that is along the axis of the bore of the MRI scanner, i.e. $B_{z}$; so, we will focus on that now but the following would apply to $B_{x}$ and $B_{y}$ as well. We can see that $B_{z}$ is the difference of two integrals,

36 $$\begin{aligned} B_{z} =&\frac{\mu_{0}}{4 \pi} \int_{V} \frac{J_{x} (y-y')}{ ( (x-x')^{2}+(y-y')^{2}+(z-z')^{2} )^{\frac{3}{2}}}\, dx'\, dy'\, dz' \\ &{}-\frac{\mu_{0}}{4 \pi} \int_{V} \frac{J_{y} (x-x')}{ ( (x-x')^{2}+(y-y')^{2}+(z-z')^{2} )^{\frac{3}{2}}}\, dx'\, dy'\, dz'. \end{aligned}$$

If we define the *x* and *y* components of a function **G** as

37a $$\begin{aligned} G_{x}&=\frac{x}{ ( x^{2}+y^{2}+z^{2} )^{\frac{3}{2}}}, \end{aligned}$$

37b $$\begin{aligned} G_{y}&=\frac{y}{ ( x^{2}+y^{2}+z^{2} )^{\frac{3}{2}}}, \end{aligned}$$

then we can define $B_{z}$ as a difference of two convolutions [34],

38 $$ B_{z}=\frac{\mu_{0}}{4 \pi} (J_{x} * G_{y} ) - \frac{\mu_{0}}{4 \pi} ( J_{y} * G_{x} ). $$

Convolution becomes multiplication in the Fourier domain [34] thus $B_{z}$ finally is calculated as

39 $$ B_{z}=\frac{\mu_{0}}{4 \pi}\mathcal{F}^{-1} \bigl\{ \mathcal{F} \{ J_{x} G_{y} \}-\mathcal{F} \{ J_{y} G_{x} \} \bigr\} , $$

where $\mathcal{F}$ is the Fourier transform [34].

## Numeric Examples and Discussion

**Fig. 2:**
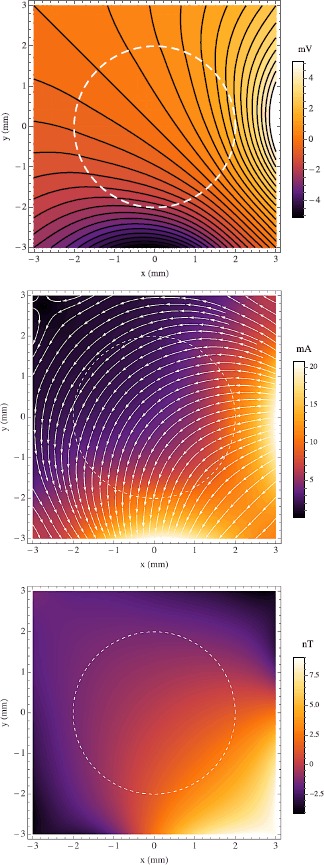
*The top*, *middle*, and *bottom images* are plots of the electric potential, current density, and magnetic flux density, respectively, in the plane $z=0$. *The dashed white line* indicates the circumference of the sphere. In the cartesian coordinates on this graph the current point source is at $(5,0)$ and the current point sink is at $(0,-5)$. The ratio $\rho_{e}/\rho_{o}=1$. In *the top graph* we show extracellular potential of the sphere of tissue amid the external potential in the conducting bath. There *the black lines* are equipotentials and *the shade of color* corresponds to the magnitude. In *the middle graph* we show the external current density in the bath and the sum of the intracellular and extracellular current densities in the sphere. The magnetic flux density shown in *the bottom graph* was calculated from the current density field

**Fig. 3:**
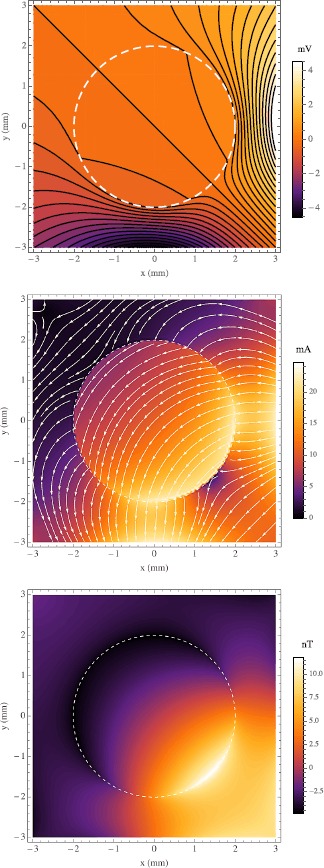
*The top*, *middle*, and *bottom images* are plots of the electric potential, current density, and magnetic flux density, respectively, in the plane $z=0$. *The dashed white line* indicates the circumference of the sphere. In the cartesian coordinates on this graph the current point source is at $(5,0)$ and the current point sink is at $(0,-5)$. The ratio $\rho_{e}/\rho_{o}=0.1$. In *the top graph* we show extracellular potential of the sphere of tissue amid the external potential in the conducting bath. There *the black lines* are equipotentials and *the shade of color* corresponds to the magnitude. In *the middle graph* we show the external current density in the bath and the sum of the intracellular and extracellular current densities in the sphere. The magnetic flux density shown in *the bottom graph* was calculated from the current density field

**Fig. 4:**
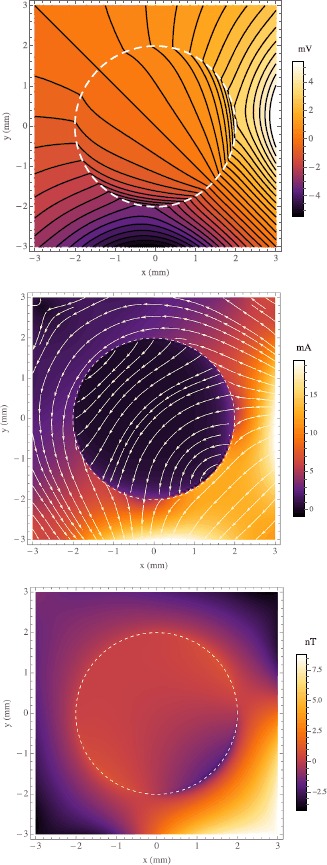
*The top*, *middle*, and *bottom images* are plots of the electric potential, current density, and magnetic flux density, respectively, in the plane $z=0$. *The dashed white line* indicates the circumference of the sphere. In the cartesian coordinates on this graph the current point source is at $(5,0)$ and the current point sink is at $(0,-5)$. The ratio $\rho_{e}/\rho_{o}=10$. In *the top graph* we show the extracellular potential of the sphere of tissue amid the external potential in the conducting bath. There *the black lines* are equipotentials and *the shade of color* corresponds to the magnitude. In *the middle graph* we show the external current density in the bath and the sum of the intracellular and extracellular current densities in the sphere. The magnetic flux density shown in *the bottom graph* was calculated from the current density field

We now demonstrate our modeling with some simple examples in which we show the effect of varying $\rho_{o}$. Let us assume the tissue radius is $a=2~\mbox{mm}$, the point current source is at $\mathbf {p}_{+}=(5,\frac{\pi }{2},0)$, and the point current sink is at $\mathbf {p}_{-}=(5,\pi,0)$. Plainly said, there is a cathode 2 mm to the right of, and an anode 2 mm directly below, a ball of tissue in an otherwise uniform ocean of artificial sea water. We choose resistivities in a range known to be biologically realistic [10], specifically $\rho_{e}=0.29~\varOmega\mbox{m}$, $\rho_{i}=0.19~\varOmega\mbox{m}$, and $\rho_{m}=0.15~\varOmega\mbox{m}$. The source and sink are positive and negative, respectively, with equal magnitude $I_{0}=1~\mbox{mA}$. The upper bound was chosen that the solution stabilized to 5 decimal places, i.e. $\mu=10$. All of these inputs are summarized in Table 1. In our first three examples we hold $\rho_{o}=\rho_{e}$, $\rho_{o}=10 \rho _{e}$, $\rho_{o}=0.1 \rho_{e}$, shown in Figs. 2, 3, and 4, respectively. The figures are squares of 3 mm so as not to include the source and sink which would obscure the behavior in and immediately surrounding the sphere. In the top graph of each figure is plotted $\phi_{o}$ and $\phi _{e}$ where they appear in the *xy* plane that includes the origin. The solid black lines are the equipotentials with the shade of color between them corresponding to the magnitude. The middle graphs show the current densities: $\mathbf {J}_{e}$ outside the sphere and $\mathbf {J}_{i}+\mathbf {J}_{o}$ inside the sphere. The arrows give the direction of the current while the color corresponds to the magnitude $|\mathbf {J}|=\sqrt {J_{x}^{2}+J_{y}^{2}+J_{z}^{2}}$. It should be noted that there is a symmetry about the plane depicted since it contains the source and sink points and the orthodrome of the sphere of tissue, thus all the current is in the plane of the page, i.e. $J_{z}=0$. In the bottom graphs we show the magnetic flux density to which $\mathbf {J}_{e}$, $\mathbf {J}_{i}$, and $\mathbf {J}_{o}$ give rise. We composed a program in MATLAB (The MathWorks, Inc., Natick, MA) that employs the fast Fourier and inverse Fourier transforms to compute $B_{z}$ from the simulated **J** data throughout a $3~\mbox{mm} \times3~\mbox{mm} \times3~\mbox{mm}$ cube whose origin is the same as the sphere’s. The **J** sampling was 13 slices, evenly spaced along the *z* axis, each containing $128 \times128$ data points. All of these results are as expected. But for the dashed white line, the sphere of tissue is indistinguishable from the surrounding bath in Fig. 2. When the interstitium has one tenth the bath’s resistivity (Fig. 3) the current can be seen to go toward the sphere resulting in a large $B_{z}$ at the sphere’s edge in the fourth quadrant of the field of view. Accordingly, when the interstitium is ten times as resistive as the bath (Fig. 4), the current flows mostly around it. In terms of the $B_{z}$ field, there appears a faint glow around the sphere’s edge, surrounding a region of relative darkness, due to this flow pattern. In both cases of $\rho_{o}\ne\rho_{e}$ the tissue is clearly visible in all three field types, *ϕ*, **J**, and $B_{z}$.

**Table 1:** **Modeling inputs**

Parameter	Value
Bath resistivity, $\rho_{e}$	0.29 *Ω*m
Intracellular resistivity, $\rho_{i}$	0.19 *Ω*m
Membrane resistance times unit area, $R_{m}$	0.15 *Ω*m^2^
Ratio of surface area to volume, *β*	20,000 m^−1^
Source and sink magnitude, $I_{0}$	1 mA
Tissue radius, *a*	2 mm
Point source position, $\mathbf {p}_{+} $	$(5,\frac{\pi}{2},0)$
Point sink position, $\mathbf {p}_{-} $	(5,*π*,0)
Summation upper bound, *μ*	10

**Fig. 5:**
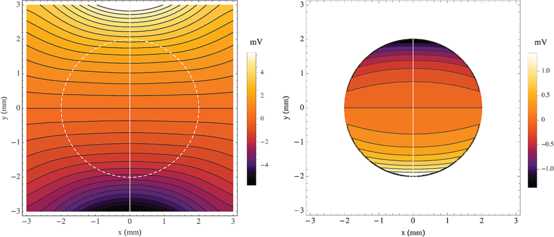
On *the left* is a contour plot of the extracellular potential of the sphere of tissue amid the external potential in the conducting bath. *The dashed white line* indicates the circumference of the sphere. On *the right* is a contour plot of the transmembrane potential within the sphere. In both graphs, *the black lines* are equipotentials and *the shade of color* corresponds to the magnitude. In the cartesian coordinates on these graphs the current point source is at $(0,5)$ and the current point sink is at $(0,-5)$. The ratio $\rho_{e}/\rho _{o}=1$

**Fig. 6:**
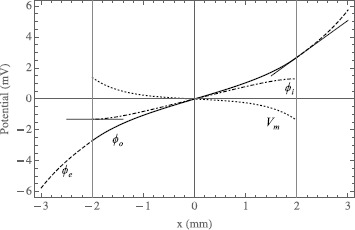
The intracellular (*dot-dashed*), extracellular (*solid*), transmembrane (*dotted*), and bath (*dashed*) potentials are shown as a function of *y*, along *the white line* where $x=0$ in Fig. 5. *The vertical lines* at $y=-2$ and 2 correspond to the radius of the sphere, $r=a$, where are also plotted the lines tangent to the curves of the potentials in the boundary conditions

As a limiting example, let us move the source point to be directly above the tissue such that $\mathbf {p}_{+}=(5,0,0)$. This is an axially symmetric problem, identical to our earlier work [35], and we get the same results, shown in Figs. 5 and 6. The white line in Fig. 5 is the horizontal axis in the plot in Fig. 6 where we have plotted $\phi _{o}$, $\phi_{i}$, $V_{m}$, and $\phi_{e}$ as a function of distance, along the segment joining the sink and source. The vertical lines at $y=-2$ and 2 indicate the extent of the tissue where it is clear by inspection that all the boundary conditions are satisfied. At the tissue radius $\phi_{o}$ (solid line) and $\phi_{e}$ (dashed line) are equal as are their slopes, and the slope of $\phi_{i}$ (dot-dashed line) is zero. Furthermore, we can see that $V_{m}$ (dotted line) is the difference between $\phi_{i}$ and $\phi_{o}$, fulfilling Eq. (3a).

In this article we set out to model the electromagnetic fields in and around a volume of neural tissue stimulated by current that is injected in close proximity to it. The geometry selected is expected in an *in vitro* MREIT scan, where an AG may be submerged in a bath of artificial seawater contained in a cylindrical sample chamber that has injection current ports on opposing sides [7]. We note that the effect of the applied external field is to simultaneously depolarize and hyperpolarize portions of the simulated tissue nearby the current sources. If a portion of tissue is sufficiently depolarized to form an action potential it may propagate throughout the tissue from these regions. It has been suggested [36] that modest depolarizations or hyperpolarizations caused by weak external currents applied to the skull are sufficient to excite or inhibit neural excitability in brain structures. More complex models of the tissue and field geometry used here may prove useful methods of exploring the mechanisms of such neuromodulation techniques. In these numeric examples we have held the source and sink points to be equidistant from the sphere of tissue, $r_{+}=r_{-}$. This gives the problem a symmetry about the lines $y=-x$ in Figs. 2, 3, and 4 and $y=0$ in Fig. 5. However, the AG is smaller than the diameter of the sample chamber; so, it will not necessarily be directly between the ports, spoiling this axial symmetry. Thus a complete three dimensional treatment of this type of problem is finally required.

From Eqs. (4a)-(4b) we can see that each region, intracellular and extracellular, has a monodomain component *ψ* that obeys the Laplace equation. Plots of this potential produce results similar to those of Rush and Driscoll [37, 38]. They solved for the electric potential in a brain from electrodes placed directly on a scalp, modeling the brain, skull, and scalp as different layers of monodomain tissues, i.e. a sphere encased in a thin shell of bone which was itself encased in a thin shell of skin. We could amend our model to include similar surrounding layers, each with its own expressions for *ϕ* and **J** and coefficients determined from the boundary conditions. The boundary conditions themselves would change, e.g. the interstitium would have continuity of potential and normal current with the skull rather than with artificial sea water. Such changes would be appropriate for a model on the scale of e.g. a dog’s head [39].

We have modeled both domains as being ohmic, i.e. their impedivities $z=\rho$ are only real valued, but *z* can be made complex by introducing a frequency dependence [34]. In their extensive literature review [40] and experimental measurements [41], Gabriel et al. show that most tissues have frequency dependent electrical properties. More recently, Bédard et al. [42] and Bazhenov et al. [43] explored frequency dependence in local field potentials. In a series of theoretical papers Bédard and Destexhe provide a general framework for modeling electromagnetic fields in brain tissue without assuming the interstitium to be purely resistive. Absent those assumptions, they developed a generalized formalism of current source density analysis with the goal of relating the extracellular potential to current sources in the tissue [44], considering monopolar sources, dipolar sources, and combinations thereof. Next they incorporated frequency dependent extracellular and intracellular impedivities, $z_{o}$ and $z_{i}$, to generalize the cable theory [11] for neurons embedded in a complex interstitium [45]. They showed that $z_{o}$ and $z_{i}$ have a non-trivial impact on the properties of neurons, e.g. voltage attenuation with distance and the spectral profile of $V_{m}$. Finally, they calculated the magnetic fields generated from a current-carrying neuron and, using superposition, a population of neurons [46]. They showed that since the electrical properties of neural tissue impact the transmembrane and axial currents of a neuron, they will necessarily also impact the magnetic fields these currents create. By contrast, in our study we have concerned ourselves with the interaction of neural tissue and an aphysiologic stimulus. The effects of this stimulus will naturally also depend upon complex tissue properties, but over a larger scale determined by the stimulus geometry. Future work should explore the impact non-ohmic impedivities have on a tissue interactions with applied external fields.

Let us now consider possible next steps to build on this first study. As well as modeling tissue properties as complex, it should also be possible to examine the transient behavior of excitable tissue using a Hodgkin–Huxley-like model [47]. These analytical models of spheres could then be validated and used to estimate the scale of changes expected in MREIT images due to different neural activity patterns.

## Figures and Tables

**Fig. 7:**
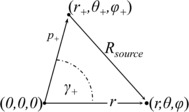
Geometry of the point current source at $\mathbf {p}_{+}=(r_{+},\theta_{+},\varphi_{+})$ in relation to the origin $(0,0,0)$ and a field point $\mathbf {r}=(r,\theta,\varphi)$, with $R_{\mathrm{source}}$ being the distance between **r** and $\mathbf {p}_{+}$

## Appendix

The potential due to the current point source, shown in Fig. 7, goes as the inverse of the distance from it which is given as

40 $$ R_{\mathrm{source}}=\vert \mathbf {r} -\mathbf {p}_{+} \vert = \sqrt{r^{2}+r_{+}^{2}-2rr_{+} \cos (\gamma_{+})}, $$

and whose inverse has the form of the generating function of the Legendre polynomial [32], allowing us to write

41 $$ \frac{1}{\sqrt{r^{2}+r_{+}^{2}-2rr_{+} \cos(\gamma_{+})}}=\sum_{\mu=0}^{\infty } \frac{ ( g_{+}^{< } )^{\mu}}{ ( g_{+}^{>} )^{\mu+1}}P_{\mu }\bigl(\cos(\gamma_{+})\bigr), $$

where $P_{\mu}$ is the Legendre polynomial of order *μ*. The spherical law of cosines [29] lets us write *γ* in terms of *θ* and *φ*,

42 $$ \cos(\gamma_{+})=\cos(\theta)\cos(\theta_{+})+\sin(\theta)\sin( \theta_{+})\cos (\varphi-\varphi_{+}), $$

which, from spherical harmonic addition theorem [48], allows us to expand $P_{\mu}$ in terms of $Y_{\mu}^{\nu}$,

43 $$ P_{\mu}\bigl(\cos(\gamma_{+})\bigr)=\frac{4 \pi}{2 \mu+1}\sum _{\nu=-\mu}^{\mu} Y_{\mu}^{\nu}( \theta,\varphi) Y_{\mu}^{-\nu}(\theta_{+},\varphi_{+}). $$

The analysis for a current point *sink* proceeds analogously. From Eqs. (19a)-(19b), the potentials for current points source and sink finally are given in spherical coordinates as

44a $$\begin{aligned} \phi_{\mathrm{source}}(r,\theta,\varphi)&=I_{0} \rho_{e} \sum_{\mu=0}^{\infty}\sum _{\nu =-\mu}^{\mu}\frac{ ( g_{+}^{< } ) ^{\mu}}{ ( g_{+}^{>} )^{\mu +1}} \frac{1}{2\mu+1}Y_{\mu}^{\nu}(\theta,\varphi) Y_{\mu}^{-\nu}(\theta _{+},\varphi_{+}), \end{aligned}$$

44b $$\begin{aligned} \phi_{\mathrm{sink}}(r,\theta,\varphi)&=-I_{0} \rho_{e} \sum_{\mu=0}^{\infty}\sum _{\nu =-\mu}^{\mu}\frac{ ( g_{-}^{< } )^{\mu}}{ ( g_{-}^{>} )^{\mu +1}} \frac{1}{2\mu+1}Y_{\mu}^{\nu}(\theta,\varphi) Y_{\mu}^{-\nu}(\theta _{-},\varphi_{-}). \end{aligned}$$
